# In vitro antioxidant activity of alginate nanoparticles encapsulating the aqueous extract of *Coccinia grandis L*.

**DOI:** 10.55730/1300-0527.3573

**Published:** 2023-06-05

**Authors:** Walimuni Nayomi Deshani DE SILVA, Anoja Priyadarshani ATTANAYAKE, Liyanage Dona Ashanti Menuka ARAWWAWALA, Desiree Nedra KARUNARATNE, Geethi Kaushalya PAMUNUWA

**Affiliations:** 1 Department of Biochemistry, Faculty of Medicine, University of Ruhuna, Galle, Sri Lanka; 2 Herbal Section, Industrial Technology Institute, Colombo, Sri Lanka; 3 Department of Chemistry, Faculty of Science, University of Peradeniya, Peradeniya, Sri Lanka; 4 Department of Horticulture and Landscape Gardening, Faculty of Agriculture and Plantation Management, Wayamba University of Sri Lanka, Kuliyapitiya, Sri Lanka

**Keywords:** Antioxidant activity, *Coccinia grandis*, encapsulation, nanoparticles

## Abstract

Bioactive compounds in medicinal plants are more susceptible to preventing oxidative stress. Encapsulation of herbal extracts has empowered the properties and characteristics of bioactive compounds. Nanoencapsulation allows the enhancement of the stability of extracts and targeted drug delivery. The present study aims to determine the antioxidant activity of alginate nanoparticles encapsulating the aqueous extract of *Coccinia grandis* L. (Family: Cucurbitaceae). The aqueous extract of *C. grandis* (AqCG) was prepared by using ultrasonication (40 °C, 20 min, 40 kHz) followed by refluxing (2½ h). The prepared AqCG (1–5 mg/mL) encapsulated alginate nanoparticles were synthesized by ionic gelation with the addition of extracts and CaCl_2_. Characterization of nanoparticles was performed via encapsulation efficiency (EE%), loading capacity (LC%), particle size (PS), scanning electron microscopy (SEM), zeta potential and Fourier transform infrared (FTIR) spectroscopy analysis. The antioxidant activity of the nanoparticles was evaluated in vitro by the ferric reducing antioxidant (FRAP) assay, 2,2-di-phenyl-1-picrylhydrazyl (DPPH) radical scavenging assay and 2,2’-azino-bis (3-ethylbenzothiazoline-6-sulfonic acid) (ABTS) radical scavenging assay. One-way analysis of variance (ANOVA) followed by Tukey’s posthoc test was used to analyze the data. Maximum LC% (3.07 ± 0.11) and average particle size (71 nm from SEM) were obtained for alginate nanoparticles encapsulated at 4 mg/mL extract concentration. The IC_50_ values for DPPH, ABTS, and FRAP were 6.49 ± 0.10 mg/mL, 0.24 ± 0.01 mg/mL, and 20.63 ± 0.28 mg Trolox equivalent/g of extract respectively for alginate nanoparticles encapsulating the AqCG. Nanoparticles have shown a significant difference in IC_50_ values compared to Trolox (p < 0.05). The successful encapsulation of the AqCG in the alginate matrix was evidenced by FTIR and SEM analysis. Encapsulation contributed to enhancing the antioxidant activity in terms of ABTS assay when compared to the AqCG. However, in vitro release and stability studies are warranted to facilitate the development of a commercially viable nanonutraceutical using alginate nanoparticles encapsulating the AqCG.

## 1. Introduction

Oxidative stress is one of the main reasons for the pathogenesis of numerous chronic diseases i.e. diabetes mellitus, asthma, Parkinson’s and Alzheimer’s disease, cancers, and atherosclerosis. Free radicals are considered key agents which exacerbate the pathogenesis of diseases [1,2]. Antioxidants are defined as substances that inhibit cell damage caused by the oxidation of molecules. During the oxidation process, free radicals are generated, and chain reactions are initiated. An imbalance of free radical production and utilization may lead to the developing oxidative stress [3]. The complex system of antioxidants in the human body are counterparts to oxidative stress, whereas antioxidants are widely used as dietary supplements. The utilization of plant antioxidants which are formulated as nutraceuticals in phytomedicine has been intensively studied targeting the early management of chronic diseases [1,4].

Since the prehistoric era, medicinal plant preparations have been used to alleviate the symptoms of diseases worldwide. Despite many attempts and the discovery of drug leads and pharmacological agents in Western medicine, medicinal plants, as a key element in many herbal drugs, continue to make a significant contribution to the treatment of chronic diseases to a great extent. However, significant attention is paid to medicinal plants due to their long-term use in traditional folk medicine based on herbals, especially in developing Asian countries such as Sri Lanka. Several herbs have been investigated for their antioxidant properties. *Coccinia grandis* L. is one of the most popular edible herbs in the Cucurbitaceae family. The antioxidant potential of *C. grandis* has been investigated through in vitro antioxidant assays, particularly 2,2-diphenyl-1-picryl-hydrazyl-hydrate (DPPH) radical scavenging assay, ferric reducing antioxidant potential (FRAP) assay and nitric oxide (NO) radical scavenging assay [5–7]. The antioxidant activity of *C. grandis* is further proved in animal models with increased oxidative stress [8,9]. Rutin, β-sitosterol, kaempferol-3-O-rutinoside, cephalandrol, quercetin-3-O-neohesperidoside, kaempferol-3-O-glucoside were isolated as potent bioactive compounds from the plant *C. grandis* and showed antioxidant potential [10,11]. The aforementioned compounds may inhibit the enzymes that generate reactive oxygen species.

Although these bioactive compounds have gained notable interest in phytomedicine, bitter taste and odor discourage the consumption of free/crude extracts. Furthermore, herbal extracts are generally sensitive to adverse conditions and can cause nutritional losses. In fact, herbal extracts can become inactive before reaching the target area/organ [12]. Therefore, it is highly necessary to enhance the quality of administration and therapeutic effect of antioxidants by addressing these limitations of herbal extracts. There is a growing consensus among researchers that the encapsulation of herbal extracts may enhance the antioxidant potential while preserving their functions as nutraceuticals [13–15].

Significant efforts have been made to synthesize herbal extracts loaded/coated nanoparticles/hydrogel beads using sodium alginate due to their nontoxicity and biocompatibility [16–18]. In the present study, sodium alginate was used as a matrix to encapsulate the aqueous extract of *C. grandis* (AqCG) due to the favorable properties of sodium alginate. Although several studies to date have focused on the antioxidant activity of AqCG, the report on the antioxidant activity of alginate nanoparticles encapsulating the AqCG has not been published yet. Therefore, the objective of this study was to optimize the concentration of AqCG to synthesize alginate nanoparticles encapsulating the AqCG, characterize alginate nanoparticles encapsulating the AqCG in terms of encapsulation efficiency (EE%), loading capacity (LC%), particle size analysis (PSA), polydispersity index (PI), zeta potential analysis, scanning electron microscopy (SEM) and Fourier transform infrared (FTIR) spectroscopy analysis and evaluate in vitro antioxidant activity of alginate nanoparticles encapsulating the AqCG by DPPH radical scavenging assay, 2,2’-azino-bis(3-ethylbenzothiazoline-6-sulfonic acid) (ABTS) radical scavenging assay and FRAP assay.

## 2. Materials and methods

### 2.1 Chemicals, reagents, and materials

Folin-Ciocalteu reagent, sodium alginate (low viscosity), sorbitan monooleate (span 80), calcium chloride, 1,1-diphenyl-2-picrylhydrazyl (DPPH), 2,2-azino-bis (3-ethylbenzothiazoline-6-sulfonic acid) diammonium salt (ABTS), Trolox, ferrous chloride, 2,4,6-tripyridyl-s-triazine (TPTZ) and sodium acetate tetrahydrate were purchased from Sigma-Aldrich Chemical Company (USA). All other reagents were selected in analytical grade.

### 2.2 Collection of plant material and authentication

The leaves of *C. grandis* were collected from the natural habitats in Galle, Southern Province. The morphology of the plant was compared with descriptions and diagrams represented in the literature. The National Herbarium of the Royal Botanical Gardens, Peradeniya, Sri Lanka, provided the service of authentication of plant material. Herbarium sheet which included the dried powdered plant leaves was conserved at Department of Biochemistry, Faculty of Medicine, University of Ruhuna, Sri Lanka under the collected number AC/NA-1.

### 2.3 Preparation of plant extracts

The leaves of *C. grandis* were dried in an oven (40 °C) and ground to obtain the powdered form of the dried leaves. The AqCG (0.06 mg/mL) was sonicated (40 °C, 20 min, 40 kHz) and then refluxed (100 °C, 2½ h). The resultant extract was filtered via filter paper (Whatman no. 1) and was freeze dried using a freeze drier (IlshinBioBase, South Korea) at −55 °C. The extract was refrigerated at 4 °C until further use.

### 2.4 Preparation of alginate nanoparticles encapsulating the AqCG

Alginate nanoparticles encapsulating the AqCG were synthesized by the ionic gelation method [19]. Briefly, sodium alginate (0.3% w/v, 100 mL) was stirred (1100 rpm) using a magnetic stirrer (VELP Scientifica, USA) in the presence of span 80 for 2 h. In order to select the optimal extract concentration based on the highest EE% and LC%, the AqCG was used in the range of 1–5 mg/mL. The AqCG was added while stirring and continued for another 2 h. To solidify the complex, CaCl_2_ (0.1 w/v %, 100 mL) was added while enhancing the stirring (1400 rpm) for 1 h [19]. The resulting solution was kept in the refrigerator overnight. The centrifugation (10,000 rpm, 45 min) was done for the refrigerated suspension to obtain the nanoparticle pellets. The pellets obtained were freeze dried (−55 °C) using a freeze dryer (IlshinBioBase, South Korea) and the weight of the formed nanoparticles was measured. Alginate nanoparticles encapsulating the AqCG were refrigerated at 4 °C until further use.

### 2.5 Characterization of alginate nanoparticles encapsulating the AqCG

The characterization of the alginate nanoparticles encapsulating the AqCG was performed in terms of the EE%, LC%, PSA, PI, zeta potential analysis, SEM, and FTIR.

The percentage of EE of the alginate nanoparticles encapsulating the AqCG was calculated using the total polyphenol content with respect to the supernatant as follows. The total polyphenol content in separated supernatants and the initial plant extracts (1–5 mg/mL) was determined using the spectrophotometric method in the presence of Folin–Ciocalteu reagent [20]. The mixture contained supernatant/initial plant extract (100 μL), 95% ethanol (100 μL), 50% Folin–Ciocalteu reagent (50 μL), distilled water (500 μL) and Na_2_CO_3_ (100 μL) were incubated at 25 °C for 1 h. The absorbance values were measured at the wavelength of 725 nm using a microplate reader (Thermo Scientific Varioskan LUX, New Zealand). A calibration curve was plotted with respect to gallic acid (5–140 μg/mL) for the determination of the polyphenol content in the supernatant and the initial AqCG (y = 0.006089 x + −0.03664 (coefficient of determination (R^2^) = 0.95). EE% and LC% were calculated as follows [21]. Calibration was performed in replicates.


$$\begin{array}{l} \text{EE }(\%)=\frac{\text{Mass of encapsulated polyphenol rich extract}}{\text{Mass of polyphenol rich extract initially introduced}}\times 100\%\\ \text{LC }(\%)=\frac{\text{Mass of encapsulated polyphenol rich extract}}{\text{Total weight of nanoparticles}}\times 100\% \end{array}$$

The alginate nanoparticles encapsulating the AqCG were subjected to PSA and zeta potential analysis. PSA measurements were taken for the resulting pellets by dispersing the pellet in distilled water using a particle size analyzer (Malvern, Zetasizer Nano ZS, UK) at a scattering angle. PSA was done using the dynamic light scattering technique (DLS). Similarly, the alginate nanoparticles encapsulating the AqCG pellet were dispersed in distilled water and the zeta potential of the nanoparticles was measured using the Zeta potential analyzer (Malvern, Zetasizer Nano ZS, UK).

The morphology (shape and diameter) of the nanoparticles was examined by SEM (Hitachi SU6600, USA). Alginate nanoparticles encapsulating the AqCG were placed in an aluminum stub. The stub was placed in the sample port of the electron microscope after coating with a gold layer and the morphology of the alginate nanoparticles encapsulating the AqCG was assessed [22].

FTIR spectra (Bruker Vertex 80, Germany) of sodium alginate powder, AqCG, blank nanoparticles, and alginate nanoparticles encapsulating the AqCG were obtained at a resolution of 4 cm^−1^.

### 2.6 Stability measurement by visual observation

Visual observation was carried out to detect the stability of alginate nanoparticles encapsulating AqCG in terms of appearance and color. Alginate nanoparticles encapsulating AqCG were placed in two Petri dishes, and one was kept at 27 °C while the other was kept in refrigerator (4 °C) for one month (28 days). Photographs of different conditions on the very first day (0 day), after one month at 27 °C and 4°C were obtained. The appearance and color of the alginate nanoparticles encapsulating AqCG were compared under different conditions.

### 2.7 Determination of in vitro antioxidant activity

The antioxidant activities of the alginate nanoparticles encapsulating the AqCG were evaluated in terms of the DPPH, ABTS radical scavenging assay, and FRAP assays.

#### 2.7.1 DPPH radical scavenging assay

The antioxidant activity of the alginate nanoparticles encapsulating the AqCG was determined based on the DPPH radical scavenging assay [23]. DPPH solution (20 mg/mL) was prepared to dissolve in methanol. Different series of AqCG and its alginate nanoparticles (1–20 mg/mL) were prepared. Methanol (90 μL), prepared AqCG and its encapsulated nanoparticles (50 μL), and the DPPH solution (60 μL) were added to the microplate. The resultant solution was incubated at 37 °C for 10 min. The absorbance values were measured at a wavelength of 517 nm. The antioxidant activity of alginate nanoparticles encapsulating the AqCG was calculated and expressed, in terms of IC_50_ (concentration of alginate nanoparticles encapsulating the AqCG at 50% inhibition). The IC_50_ value of the alginate nanoparticles encapsulating the AqCG was compared with the standard compound Trolox [24].

#### 2.7.2 ABTS radical scavenging assay

The ABTS scavenging activity of the alginate nanoparticles encapsulating AqCG was measured spectrophotometrically at 734 nm, as previously described with slight modifications [14]. The stock solution of ABTS (4 mg/mL) was prepared at a concentration of 2.5 mM of potassium persulfate solution. The stock solution was incubated for 16 h at 37 °C in the dark. The ABTS radical cation (ABTS^+^) was formed upon incubation. Then, the ABTS^+^ solution was diluted (1:7) with phosphate buffer (50 mM, pH 7.4). Alginate nanoparticles encapsulating the AqCG were prepared at various concentrations (0.0625–1.0 mg/mL). The buffer (110 μL), prepared nanoparticles (50 μL), and ABTS^+^ (40 μL) were added to the microplate. Then, it was incubated at 37 °C for 10 min. After a period of incubation, the absorbance was measured at 734 nm. Trolox was used as a reference compound and the ABTS radical scavenging activity was calculated as IC_50_ [23].

#### 2.7.3 FRAP assay

The reducing power of the alginate nanoparticles encapsulating the AqCG was performed using a standard Trolox curve (0.03–1 mg/mL) (y = 3.244 x + 0.02069 (R^2^ = 1)). The FRAP reagent was prepared using acetate buffer (300 mM, pH 3.6, 10 mL), 10 mM solution of 2, 4, 6- tripyridyl-s-triazine (TPTZ) in HCl (40 mM, 1 mL) and FeCl_3_ solution (20 mM, 1 mL). Alginate nanoparticles encapsulating AqCG (1 mg/mL) react with FRAP solution and then absorbance was measured at 600 nm. The reducing power of alginate nanoparticles encapsulating the AqCG was expressed as milligrams of Trolox equivalents/g of nanoparticles [25].

### 2.7 Statistical analysis

The results are reported as mean ± SD for the measurements. One-way analysis of variance (ANOVA) with posthoc test, Tukey was used to analyze data. p < 0.05 was considered statistically significant.

## 3. Results

### 3.1 Formation and characterization of alginate nanoparticles encapsulating AqCG

**Figure 1:**
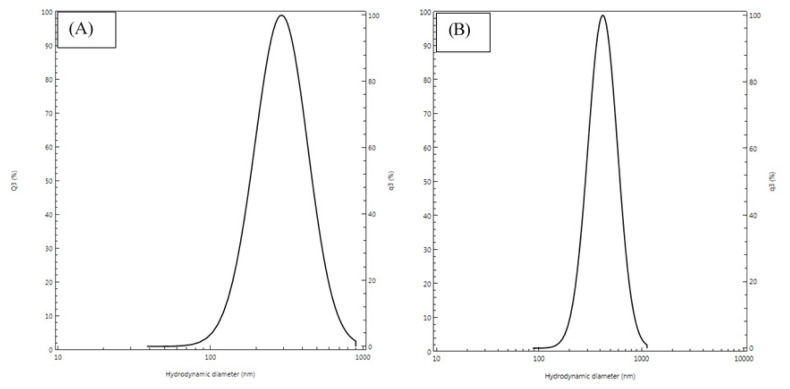
Particle size distribution curves (A) blank alginate nanoparticles, (B) alginate nanoparticles encapsulating the AqCG (4 mg/mL) Q3(%); intensity.

**Figure 2:**
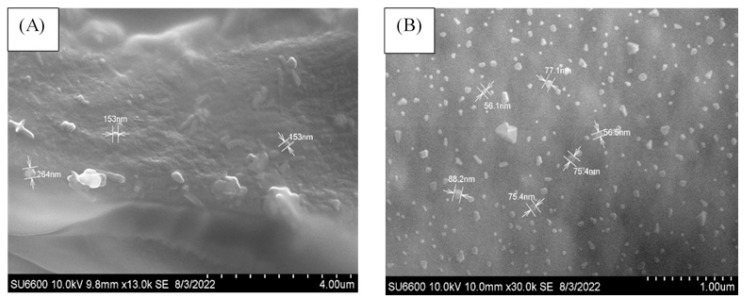
SEM images (A) blank alginate nanoparticles, (B) alginate nanoparticles encapsulating the AqCG (4 mg/mL).

Preliminary tests were carried out using different concentrations of the AqCG in order to select the optimal concentration for the synthesis of alginate nanoparticles encapsulating the AqCG. Table 1 shows the EE%, LC%, particle size (PS), and PI at different concentrations of the AqCG. The distribution curves of blank alginate nanoparticles and alginate nanoparticles encapsulating the AqCG at 4 mg/mL concentration are presented in Figures 1A and 1B, respectively. Based on the EE% and LC% results, the AqCG at a 4 mg/mL concentration was selected to synthesize the alginate nanoparticles encapsulating the AqCG. The zeta potential of the alginate nanoparticles encapsulating the AqCG was −21.0 ± 4.0 mV. The average PS and average zeta potentials of the blank nanoparticles were 287 ± 86 nm (through DLS) and −12 ± 4.1 mV respectively. The 50% alginate nanoparticles encapsulated AqCG showed 391 ± 75 nm diameter based on the results of PS through the DLS technique. The alginate nanoparticles encapsulating AqCG (4 mg/mL) were subjected to SEM analysis. Figure 2A shows the mean diameter of the blank nanoparticles as 190 nm and Figure 2B shows the mean diameter of the alginate nanoparticles encapsulating the AqCG as 71 nm.

**Figure 3:**
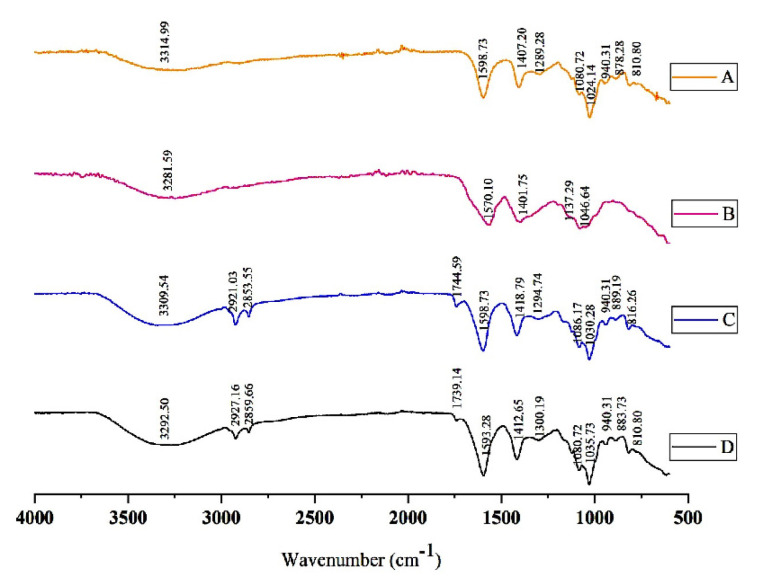
FTIR spectra of (A) sodium alginate powder, (B) AqCG, (C) blank nanoparticles, (D) alginate nanoparticles encapsulating the AqCG.

The FTIR spectra of sodium alginate powder (Figure 3A), AqCG (Figure 3B) blank nanoparticles (Figure 3C), and alginate nanoparticles encapsulating AqCG (Figure 3D) showed several substantial peaks in a wave number region of 3315–3290, 1600–1570, 1420–1400, 1040–1020 cm^−1^ reflecting O-H^−^, COO^−^ (asymmetric), COO^−^ (symmetric) and C-O-C stretching, respectively. The FTIR spectra of blank nanoparticles (Figure 3C) and alginate nanoparticles encapsulating the AqCG (Figure 3D) showed new peaks in the range of 2850–2930 cm^−1^ which did not appear in the sodium alginate powder indicating that the cross-linked reaction occurred between pure alginate and CaCl_2_. Furthermore, there are several positional changes and relative shifts in the intensity distribution in the spectrum of blank alginate nanoparticles and alginate nanoparticles encapsulating the AqCG (Figures 3C and Figure 3D). Furthermore, when compared to the AqCG (Figure 3B), the absorption bands observed in the region of 750–1750 cm^−1^ became more intense and narrower in the spectrum of alginate nanoparticles encapsulating the AqCG (Figure 3D). The peak of OH^−^ at blank nanoparticles (3309.54 cm^−1^) had shifted to a lower wave number (3292.50 cm^−1^) after the formation of alginate nanoparticles encapsulating AqCG. The position of the asymmetric and symmetric stretching peak of the COO^−^ of the blank nanoparticles (Figure 3C) (1598.73 cm^−1^ and 1418.79 cm^−1^) had also changed with the AqCG encapsulation (Figure 3D) (1593.28 cm^−1^ and 1412.65 cm^−1^) and the intensity of COO^−^ stretching peaks has improved upon encapsulation. In addition, the characteristic peak at 1744.59 cm^−1^ in blank alginate nanoparticles (Figure 3C) representing the stretching of protonated COO- (COOH) group was shifted to 1739.14 cm^−1^ in alginate nanoparticles encapsulating the AqCG (Figure 3D).

### 3.2 Measurement of stability by visual observation

**Figure 4:**
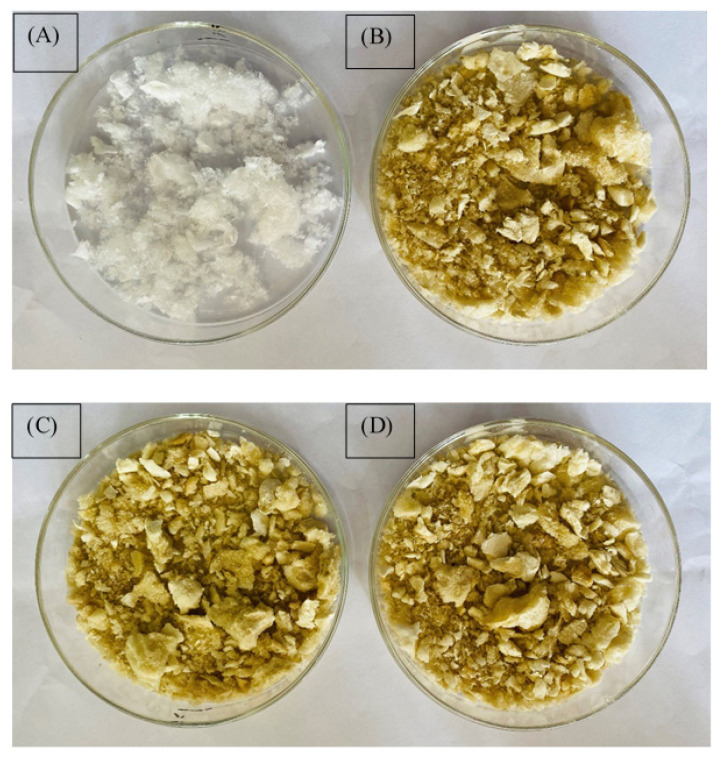
Photographs of (A) blank alginate nanoparticles, (B) alginate nanoparticles encapsulating AqCG at 0-day, (C) alginate nanoparticles encapsulating AqCG after one month at 27 °C, (D) alginate nanoparticles encapsulating the AqCG after one month at 4 °C.

Figure 4A shows the physical appearance of blank alginate nanoparticles. Figures 4B–4D show the physical appearance of alginate nanoparticles encapsulated AqCG at 0-day, at 27 °C after one month and at 4 °C after one month, respectively. The blank alginate nanoparticles are white in color, whereas the color changed into brown upon encapsulation. However, there were no changes in both appearance and color after one month at both 27 °C and 4 °C.

### 3.3 Antioxidant activity of alginate nanoparticles encapsulating the AqCG

The antioxidant activity of the AqCG and the alginate nanoparticles encapsulating the AqCG is presented in Table 2. Interestingly, the ABTS radical scavenging activity of alginate nanoparticles encapsulating the AqCG was improved to 17.24% with respect to the AqCG. Based on statistical analysis, alginate nanoparticles encapsulating the AqCG showed significant nonspecific differences with respect to both Trolox and the AqCG.

## 4. Discussion

There is a growing interest in herbal nutraceuticals with potential antioxidant activity that can be used in clinical practice. This is due to the presence of pharmacologically active compounds in the herbs. It is evidenced that health promoting effects of herbal extracts could be enhanced with the nanoencapsulation [26,27]. Nanocarriers simultaneously potentiate the action of phytoconstituents, minimize their degradation, and protect bioactive compounds from undesirable conditions. Further, it is important to preserve active compounds until consumption [17]. Therefore, there should be an effective nanocarrier with biodegradable, nontoxic, nonimmunogenic, and biocompatible with the host. These favorable properties are present in alginate and were used to encapsulate AqCG in this study. The separation of alginate into its monomeric uronic acids is achieved with the partial acid hydrolysis of alginate, while it depends on the pH of the solution. The pH of AqCG (6.72) avoids the partial acid hydrolysis of alginate during the formation of alginate nanoparticles leading to proper encapsulation of AqCG to the alginate matrix [28].

Based on the results, the EE% and LC% of alginate nanoparticles encapsulating the AqCG were improved when increasing the concentration of the AqCG. In the present study, EE% was calculated based on the consideration of encapsulation of polyphenols present in the AqCG. When interactions such as hydrogen bonding through polyphenols in the AqCG and alginate are optimal, the highest EE% was achieved. With the further increase of the concentration of AqCG, the lack of enough carboxyl groups in alginate restricts the formation of alginate nanoparticles encapsulating the AqCG, thus the EE% was decreased [29]. Variation of EE% data is based on the agreement based on the encapsulation of the protein hydrolysate extract from *Ziziphus jujube* Mill. (Family: Rhamnaceae) seed [14].

The selection of suitable solvents for effective extraction is important to maximize the number of target compounds. However, water was selected as the solvent in the present study because of its lack of toxicity and relevance in medicinal and nutritional applications. In fact, water is used for the decoctions prepared by traditional medicine practitioners [13]. Further, water could facilitate the extraction of hydrophilic antioxidants from the herbs. Despite the determination of EE% and LC%, the analysis of nanoparticle morphology, PS, and PI is also important to characterize nanoparticles. PI is used to measure the average uniformity of nanoparticles. PI values less than 0.1 are considered as a homogenous (monodisperse) population. However, higher PI values were obtained in our study, indicating the broad particle size distribution. The larger PI can occur due to agglomeration or aggregation of samples during synthesizing or analysis [30]. In this case, proper alteration of the stirring rate, the ratio of matrix and encapsulant, reaction temperature, and amount of surfactant facilitates the formation of monodisperse formulations [31].

SEM analysis was used to visualize and to obtain direct information on surface morphology, particle size distribution, and shape of the nanoparticles as well as to confirm the effect of AqCG encapsulation on the aforementioned parameters [18]. SEM analysis has shown approximately 71 nm for alginate nanoparticles encapsulating AqCG, while DLS has shown it as 391 ± 75 nm. Different particle preparation in the analysis is one of the reasons for this discrepancy [32]. Indeed, freeze-dried alginate nanoparticles encapsulating the AqCG were used for SEM analysis and a pellet of alginate nanoparticles encapsulating the AqCG dispersing distilled water was used for particle size analysis through the DLS technique. Of the two techniques, the DLS technique has several pitfalls. Dust particles and small amounts of large aggregates, in addition to distinctly smaller nanoparticles, can affect the results of the particle size [32]. Furthermore, the DLS technique is sensitive to the presence of larger particles in a sample, since the intensity of the scattered light depends on the particle size. According to the Rayleigh approximation, the intensity of the scattered light is proportional to the sixth power of the particle diameter. Therefore, even in a small number of large particles in an alginate nanoparticle encapsulated, AqCG can dominate a higher mean diameter [32,33].

Successful encapsulation of the AqCG in the alginate matrix was evidenced by positional changes and changes in peak intensity in the FTIR spectra. A broad absorption band at 3314.99 cm^−1^ in sodium alginate (Figure 3A) was attributed to the stretching of hydroxyl groups of alginate nanoparticles which had shifted to 3292.50 cm^−1^ in the case of AqCG encapsulation (Figure 3D) [34,35]. Further, the enhancement of the intensity of the adsorption band in the spectra of alginate nanoparticles encapsulating the AqCG indicates the modification or possible interaction between the AqCG and alginate nanoparticles. The differences in peak intensity of the C–O–C stretching peak of sodium alginate and blank nanoparticles specify the ionic bond between the calcium ion and the carboxyl groups of the sodium alginate [12]. The pKa value of alginate lies between 3.4 and 4.4. The alginate biopolymer is present as dissociated carboxylate ions around pH 5 facilitating the crosslinking with calcium ions. However, under a mild acidic medium and when the pH of the alginate solution is below the pKa of the carboxylic acid, the carboxylic acid could be in its protonated state [36]. In this context, the appearance of peaks at 1744.59 cm^−1^ and 1739.14 cm^−1^ in blank alginate nanoparticles (Figure 3C) and alginate nanoparticles encapsulating the AqCG (Figure 3D) is due to the stretching of protonated COO^−^ (COOH) group in calcium alginate [37]. However, the changes in the FTIR spectrum and differences in peak intensity have supported the work done by Katuwavila et al. [19] on ferrous-loaded alginate nanoparticles, Jayapal and Dhanaraj [37] on exemestane loaded alginate nanoparticles for cancer treatment, and Pasukamonset et al. [12] on alginate-based encapsulation of polyphenols comprised of *Clitoria ternatea* L. (Family: Fabaceae) petal flower extract. The enhancement of the intensity of the adsorption bands is also responsible to highlight the successful encapsulation of AqCG into the alginate matrix.

The physical appearance and color of the nanoparticles can be considered preliminary parameters of nanoparticles. As proven in the stability experiment, a change in the color of alginate nanoparticles encapsulating AqCG appeared upon encapsulation of AqCG. However, the consistency of physical appearance and color of alginate nanoparticles encapsulating AqCG after one month at both 27 °C and 4°C revealed the stability of formulated alginate nanoparticles under different conditions.

Antioxidant activity is governed based on the redox potential of an antioxidant. The DPPH assay has often been used to measure antioxidant potential because it is more rapid, inexpensive, and simple. DPPH is distinguishable as a stable free radical, while a deep violet color is given with delocalization. In the presence of an antioxidant, DPPH captures the hydrogen atom from the antioxidant and converts it to its reduced form as 2-diphenylpicrylhydrazin, indicating the yellow product. The number of reduced DPPH molecules could be proportionate with the available hydroxyl groups [5,38]. In this study, the antioxidant activity of AqCG decreased with encapsulation, while a similar phenomenon was observed for protein hydrolysate-encapsulated calcium alginate beads within 0–10 days of storage [14].

The ABTS assay can be used for both lipophilic and hydrophilic antioxidants. The ABTS^+^ radical is generated by the reaction of potassium persulfate and ABTS salt. The same as for DPPH, the ABTS radical is reduced with the acceptance of hydrogen from polyphenols. However, the antioxidant activity of alginate nanoparticles encapsulating the AqCG exhibited better inhibitory activity for ABTS radical scavenging assay than DPPH radical scavenging assay due to the high sensitivity of ABTS assay towards representing antioxidant activity. In fact, the ABTS assay has faster reaction kinetics and a high response to antioxidants, and this could explain the obtained results [39]. In fact, the high hydrophilic antioxidants present in the alginate nanoparticles encapsulating AqCG could be better reflected by the ABTS assay than by the DPPH assay [40].

The ability to reduce ferric ions and the 2,3,5-triphenyl-1,3,4-triaza-2-azoniacyclopenta-1,4- diene chloride complex by antioxidants is measured using the FRAP assay [41]. The alginate matrix is pH responsive where it shrinks at low pH, therefore encapsulants such as antioxidants are preserved. Shrinkage of the polymer chain may decrease the ability of polymer swelling. Furthermore, alginate has the ability to form a compact acid gel structure at low pH, restricting the release of encapsulant from the alginate matrix [19,42]. In this context, alginate nanoparticles encapsulating the AqCG resulted in a lower FRAP value than its free crude extract. However, Umamaheswari and Chatterjee [5] revealed that various phytochemicals such as flavonoids, tannins, saponins, phenols, and terpenoids might attribute to the antioxidant activity. It has been proven that the aforementioned phytochemicals are much more capable of reacting with free radicals, terminating radical reactions via converting free radicals to stable compounds in plant extract encapsulating nanoparticles.

According to previous studies on plant extract-loaded alginate nanocarriers, polyphenol is one of the major constituents that is effectively loaded into the alginate matrix. This is supported by Chan et al. [43], Ćujić et al. [18], Pasukamonset et al. [12], Noor et al. [44]. For instance, Pasukamonest et al. [12] synthesized alginate microbeads with the encapsulation of phenolic extracts of *Clitoria ternatea* L. (Family: Fabaceae) through the extrusion method. It is revealed that polyphenols, mainly flavonoid, and anthocyanin, were encapsulated successfully and characterized [12]. In addition, alginate microspheres of polyphenols in the presence of *Piper betle* L. (Family: Piperaceae) were successfully prepared by Noor et al. [44]. However, the previous phytochemical analysis revealed that AqCG is rich in polyphenolic compounds, alkaloids, flavonoids, saponins, and sterols [45]. On the basis of the above disclosure, it can be assumed that polyphenols in AqCG have successfully encapsulated into the alginate matrix. Furthermore, the Folin-Ciocalteu method which was performed to determine the efficiency of encapsulation also proved the successful encapsulation of polyphenols in the alginate matrix. However, the antioxidant activity of alginate nanoparticles that encapsulate AqCG may be high when a mixture of secondary metabolites is in consideration rather than encapsulating a single compound [43].

## 5. Conclusion

The AqCG was effectively encapsulated in an alginate matrix with the highest percentage of LC (3.07 ± 0.11), at the extract concentration of 4 mg/mL and was evidenced by SEM and FTIR analysis. The results of the antioxidant activity demonstrated that the encapsulation in AqCG contributed to decreased antioxidant potential by DPPH and FRAP assays whereas encapsulation contributed to enhancing the antioxidant activity in terms of ABTS assay when compared to the AqCG. This is the first scientific evidence of the antioxidant activity of alginate nanoparticles encapsulating the AqCG. However, further studies are required on the long-term stability of *C. grandis* extract nanoparticles and in vitro release prior to the development of commercially viable nanonutraceuticals with potent antioxidant activity.

## Figures and Tables

**Table 1:** The percentages of EE, LC, PS and polydispersity index (PI) under different concentrations of AqCG and alginate nanoparticles encapsulating the AqCG.

Extract concentration (mg/mL)	EE%	LC%	PS (nm)	PI
1	61.28 ± 0.95	0.94 ± 0.18	456 ± 150	0.88
2	27.00 ± 0.56	1.02 ± 0.02	538 ± 177	1.05
3	40.23 ± 0.25	1.89 ± 0.21	456 ± 159	0.88
4	56.66 ± 0.56	3.07 ± 0.11	391 ± 75	0.97
5	39.60 ± 0.29	2.07 ± 0.20	158 ± 55	0.92

EE%, encapsulation efficiency; LC%, loading capacity; PS, particle size; PI, polydispersity index.

**Table 2:** Bioactive properties of AqCG and its nanoparticles.

Antioxidant assay	AqCG	AqCG encapsulated alginate nanoparticles	Standard compound Trolox
DPPH (IC_50_ (mg/mL)	0.57 ± 0.01^a^	6.49 ± 0.10^ab^	0.02 ± 0.00
ABTS (IC_50_ (mg/mL)	0.29 ± 0.01^a^	0.24 ± 0.01^ab^	0.03 ± 0.00
FRAP (mg Trolox equivalent/g of extract)	93.18 ± 0.95	20.63 ± 0.28	

DPPH, 2,2-diphenyl-1-picryl-hydrazyl-hydrate assay; ABTS, {2,2’–azinobis-(3-ethyl-benzothiazoline-6-sulphonic acid)} assay; FRAP, ferric reducing antioxidant power. Result represented as mean ± SD, (a) significant difference with respect to Trolox, (b) significant difference with respect to the aqueous extract of *C. grandis*; p < 0.05.
